# Impact of Short-Term Intensive-Type Cognitive Behavioral Therapy Intervention on Internet Addiction among Chinese College Students: A Randomized Controlled Trial

**DOI:** 10.3390/ijerph19095212

**Published:** 2022-04-25

**Authors:** Wenjie Yang, Wenyan Hu, Nobuaki Morita, Yasukazu Ogai, Tamaki Saito, Yan Wei

**Affiliations:** 1 The Mental Health Center, Yunnan University, Kunming 650091, China; niujiazu2022@126.com; 2 Department of Social Psychiatry and Mental Health, Faculty of Medicine, University of Tsukuba, Tsukuba 305-0006, Japan; nobuakim@nifty.com (N.M.); ogai.ys@md.tsukuba.ac.jp (Y.O.); hhd02063@gmail.com (T.S.); 3 Mental Health Education Center for College Students, Zhejiang Gongshang University, Hangzhou 310018, China; 4 Faculty of Health and Sport Sciences, University of Tsukuba, Tsukuba 305-0006, Japan; wy1930380@gmail.com

**Keywords:** group intervention program, internet addiction, strengths perspective, cognitive behavioral therapy, randomized controlled trial

## Abstract

The object of this study is to examine the effects of a short-term intensive-type Cognitive Behavioral Therapy (CBT) intervention to prevent internet addiction among Chinese college students. We conducted a randomized controlled trial applying a group counseling intervention program based on CBT. Data included 21 participants in the intervention group and 22 participants in the control group. The results showed that the intervention program reduced college students’ internet addiction symptoms and procrastination and improved their sense of coherence. Regarding the sustained effect, internet addiction symptoms decreased and perceived social support from significant others improved in college students. However, the intervention program did not significantly reduce their average daily internet use time and psychological stress. Overall, this study developed a short-term intensive-type intervention program based on CBT theory, which is complementary for Chinese college students with internet addiction.

## 1. Introduction

The flourishing of the internet is one of the most prominent features of the information society. In recent years, access to the internet has become widespread with the rapid development of smartphones. The number of internet users reached approximately 4.39 billion worldwide by 2019 [1], and internet usage in developing countries increased from 7.7% to 45.3% between 2005 and 2018 [2]. According to recent data released by the China Internet Network Information Center [3], the number of Chinese internet users has reached 1.011 billion, and the internet penetration rate reached 71.6% by June 2021, more than one-fourth of whom were students. The percentage of internet users using smartphones to access the internet was as high as 99.6%, with 26.9 h of internet access per person per week. Nowadays, people’s daily lives have become inseparable from the internet, and the internet is widely used by college students for multiple purposes, including learning, entertainment, and communication [4].

In mainland China, college students have relatively less supervision from parents and teachers than elementary and middle school students and have more preference and capability to follow digital device updates, but have less self-control than adults [5]. Easy and regular access to the internet may foster excessive internet use, which has proven to be a potential risk factor for the development of Internet Addiction (IA) [4,6]. Research has shown that college students aged 18 to 25 are vulnerable to addictive behaviors [7]. Young (1998) defined IA as “an impulse control disorder unaffected by addictive substances” [8]. Kwok et al. (2017) found that IA results in various negative effects, including sleep deprivation, lack of exercise, eye discomfort, stiff shoulders, cyberbullying victimization, and debt problems [9]. A survey of 8098 Chinese college students showed that 21.4% of IA students had suicidal thoughts, significantly higher than 5.6% of non-IA students. IA students were also at risk for anxiety and depression, with a prevalence of 43.7% and 37.5%, respectively [10]. Therefore, it is important to take appropriate measures to prevent Chinese college students from IA.

### 1.1. Prevention of Internet Addiction

The prevalence of IA among college students is 11.3% in China [11], and it is still increasing [12,13]. The International Classification of Diseases 11th classifies internet users into four layers [14]. From the first to the fourth layers are “Healthy user”, “Hazardous user”, “Internet addiction disorder”, and “Complication & Comorbidity”. Prior prevention research on IA focuses on early detection and treatment of the second and the third layer internet users and attempts to conduct appropriate intervention programs to prevent them from becoming severely addicted. Vondráčková and Gabrhelík (2016) reviewed 108 studies and suggested that for those at high risk for IA, specific skills to prevent IA fall into four basic areas, including skills related to (a) internet use, (b) coping with stress and emotions, (c) interpersonal relationships, and (d) daily life routines [15].

### 1.2. Interventions for Internet Addiction

Group counseling, CBT, reality therapy, psychological health education, anthropological methods, camp therapy, family therapy, self-help groups, sports therapy, acupuncture, pharmacotherapy, and comprehensive intervention have been used as prevention or intervention programs for IA [16]. A meta-analysis of randomized controlled trial (RCT) studies showed that group counseling and CBT are the most widely used methods to reduce IA [17].

Group counseling aims to provide psychological help and guidance in groups, where helpers form target groups based on the similarity of participants’ problems. It also addresses common developmental problems and psychological disorders of the group members through joint discussion, training, and guidance [18]. Huang et al. (2015) conducted a meta-analysis of 11 group counseling intervention studies for IA among Chinese college students and showed that group counseling is effective in enhancing the communication skills of students with IA and decreasing their obsessions and anxiety symptoms [19]. In addition, another meta-analysis found that group counseling intervention programs were effective in reducing four aspects of IA, including time management, interpersonal and health issues, tolerance, and compulsive online use [17].

The cognitive-behavioral model proposed by Davis (2001) points out that maladaptive cognitions are the proximal causes of pathological internet use. Maladaptive cognitions include distorted perceptions of oneself and the outside world, such as self-doubt, low self-efficacy, negative self-esteem, or the idea that people can only be respected on the internet [20]. In addition, individuals’ psychopathology (i.e., depression, social anxiety) and stressors from the external environment, such as families and school, are considered distal causes of IA [20]. CBT intervention programs for IA developed based on the cognitive–behavioral model consist of identifying the individual’s maladaptive cognitions, emotional and behavioral factors that trigger addiction, restructuring cognitions, learning appropriate behavioral skills, and receiving social support. Meta-analysis indicated that CBT programs showed positive changes in depression, anxiety, aggression, somatization, social anxiety, fearful anxiety, paranoid ideation, and psychoticism in individuals with IA [17]. However, Malinauskas and Malinauskiene (2019) conducted a meta-analysis that did not conclude that the CBT intervention group had a significant effect on the severity of IA, although CBT was the most used compared to other psychotherapies [21].

### 1.3. Interventions for Internet Addiction among Chinese College Students 

For more than a decade, researchers have been developing various CBT group counseling intervention programs and examining their effectiveness among Chinese college students with IA. For example, Bai and Fan (2007) performed an intervention study of 48 college students with IA tendencies, 24 each in the intervention and control groups. A group counseling intervention program based on CBT was implemented for the intervention group, while the control group received no intervention. The results showed that in the post-intervention and 6-week follow-up surveys, the intervention group reported significantly lower IA scores than the control group [22]. Li and Huang (2017) performed an intervention study of 60 poor college students with IA. The intervention and control groups each consisted of 30 participants. Compared to the control group, the intervention group showed positive changes in self-esteem, mental health status, depression, and anxiety, as well as symptoms of IA after the intervention, and these effects were sustained for 2 months [23]. 

### 1.4. The Present Study

There are some issues that have not been adequately examined in previous intervention studies on IA. Firstly, the prevalence of IA among college students is becoming more pronounced with smartphones, and the prevalence of IA among female students is also gradually increasing [24]. Most intervention programs to date have targeted IA on computer terminals; therefore, programs with mobile device-based content are needed for IA prevention. In addition, previous intervention studies have included a high proportion of male participants and even exclusively male participants [25], which may create bias in the accuracy of the study results.

Moreover, most intervention programs on IA to date have focused on the risk factors for IA, attempting to change problem behaviors of college students with IA through interventions such as alternative behaviors, skills training, and even cognitive restructuring. Few studies have considered “problem behaviors” from a positive perspective and focused on college students’ own strengths and resilience. Previous intervention programs neglect to guide college students to take a broad perspective on current situations by reflecting on the meaning of life. Furthermore, rigorous and high-quality RCT trials are still rare due to various limitations in the previous study design [26]. Most of the previous studies have involved intervention programs lasting eight or more sessions and several weeks, and no RCT studies have been found to examine the effects of short-term intensive-type intervention programs.

To address the above research gaps, this study aims to examine the effects of a short-term intensive-type CBT intervention with the purpose of preventing IA among Chinese college students. The results of this study will provide a basis for developing preventive education for IA among Chinese college students and will also offer new options for psychological health education.

## 2. Methods

### 2.1. Study Design

This was a randomized controlled trial comparing an intervention group with a control group. After informed consent was given to participants who met the selection criteria, the intervention group received an intervention program in addition to a training course on IA, while the control group received only the training course on IA. Data were collected three times for both groups: pre-intervention, post-intervention, and one month later.

### 2.2. Participants 

#### 2.2.1. Selection Criteria

Students who scored above the cut-off value of 50 on Young’s Internet Addiction Test (YIAT); Students in their second year of college or above who have taken the required course of college psycho-health education in their first year; Students who understand the purpose of this study and can give informed consent to participate in this study; Students who are available to participate during the determined group intervention time frame.

#### 2.2.2. Exclusion Criteria

Students who are undergoing treatment for mental disorders; Students who are receiving other counseling or group activities related to IA. 

#### 2.2.3. Dropout Criteria 

After the intervention program started, the individual requested to withdraw from the program; The individual had difficulty continuing to participate in this program due to severe changes in his/her physical and mental conditions; Absence from at least two of the five intervention sessions.

#### 2.2.4. Basis for Setting Sample Size 

We performed a preliminary analysis using G*Power 3.1.9.7 software (Kiel University, Kiel, Germany), taking into account the two-way analysis of variance with correspondence. Effect size f = 0.25 (Medium); Significance level = 0.05; Power (1−β) = 0.80. Under these conditions, the sample size was calculated to require a total of at least 28 participants (14 each in the intervention and control groups). The study was set up with a total size of 44 participants to account for dropouts in follow-up.

#### 2.2.5. Method of Recruiting

Study participants were recruited in two ways: by posting recruitment posters at the university and by recommendations from tutors. The participants were divided into an intervention group (group A) and a control group (group B) and were randomized separately for males and females, with gender as a stratification factor to avoid differences in gender proportions between the intervention and control groups. In this case, the “Randomized Stratified Substitution Block Allocation Table” prepared in advance was used.

**Figure 1:**
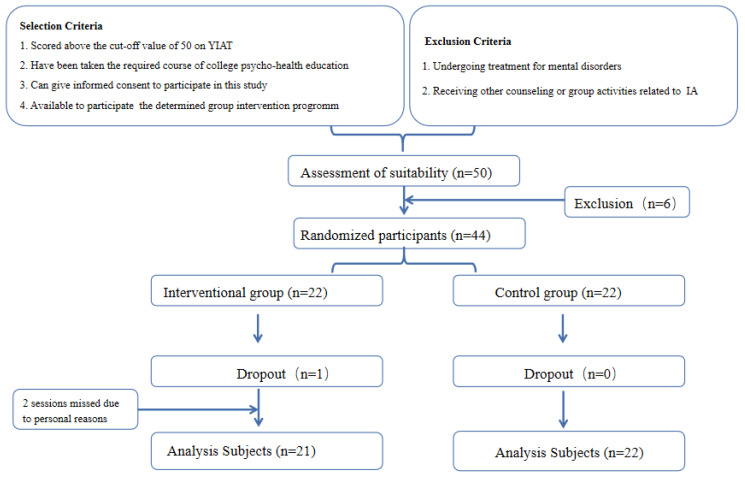
Flowchart of Randomized Controlled Trial Participants.

Participants were recruited in December 2020. The flowchart of the study participants is presented in Figure 1. A total of 50 college students were eligible to participate in this study, but 6 students were excluded due to personal reasons. As a result, 44 participants were eligible for random assignment, 22 in the intervention group and 22 in the control group. Of those who participated in the intervention program, 1 intervention group participant was excluded because she missed 2 sessions, and a total of 43 participants, 21 in the intervention group and 22 in the control group were finally included in the analysis. The study was approved by the ethics review committee of the university where the authors are affiliated.

### 2.3. Intervention Program Contents

#### 2.3.1. Intervention Program Implementers

We employed a group counseling intervention. The study was conducted under the leadership of two group leaders (both certified psychotherapists by the Japanese Health Counseling Association and certified psychological counselors by China) and with the assistance of four assistants (graduate students in psychology).

#### 2.3.2. Theory and Techniques of the Intervention Program

##### CBT Theory

Yang et al. (2021) have identified risk factors that contribute to IA and protective factors that reduce IA in previous studies [27]. Based on the cognitive–behavioral model of Davis [20], we developed an intervention program focusing on learning problem-solving skills, restructuring social support cognitions, and promoting students’ awareness of alternative behaviors.

##### Group Counseling Techniques

The plan for group counseling includes three phases: an initiation phase, a work phase, and a termination phase [18]. In this study, the initiation phase was designed to create a group environment, help members get to know each other, provide a sense of security and belonging, and create interests and expectations to participate in group activities. Next, the work phase was designed to help members increase their affirmation of self and others through special games, discussions, and growth experience sharing, to make them aware of their own way of life, and to use group counseling as a place to practice so that members can apply what they learn in their daily lives. Finally, the termination phase was intended to allow members to reflect on each session, share their own impressions and takeaways, and cope with feelings of separation through mutual encouragement.

##### Single Session Counseling Model Philosophy

The Single Session Counseling Model (SSCM) is a short-term, intensive counseling model that not only incorporates the professional therapeutic perspective of Western culture (i.e., positive psychology) but also explores professional therapeutic perspectives from a Chinese cultural perspective [28]. The overall goals of SSCM are divided into three dimensions. The surface goal focuses on solving the clients’ specific problems; the middle goal focuses on improving the clients’ lifestyle, and the deep goal focuses on the clients’ discovery of the meaning and power of life as the key to counseling success. In this process, the counselor does not assume a professional role but rather respects the clients’ existential value, free will, and search for meaning in life in order to achieve the deeper goal of discovering the meaning and power of life and trusts in their ability to make decisions actively and constructively [28]. Our study incorporated the SSCM philosophy.

##### Integration of Psychotherapy Techniques

The intervention program consisted of five themes, with three to four different group activities for each theme. The main group activities were developed by the researchers using group counseling techniques, but we also established our own activities using different psychotherapy techniques to achieve specific thematic goals. For example, “metaphorical story” is an activity that applies the hypnotherapy techniques developed by American psychiatrist Milton Erickson, and the “Ikiru” film viewing program was developed based on the philosophy of existential therapy proposed by American psychiatrist Irvin D. Yalom. Furthermore, “stress temperament coaching” is a technique of the Structured Association Technique [29] proposed by Japanese psychologist Tsunetugu Munakata.

**Figure 2:**
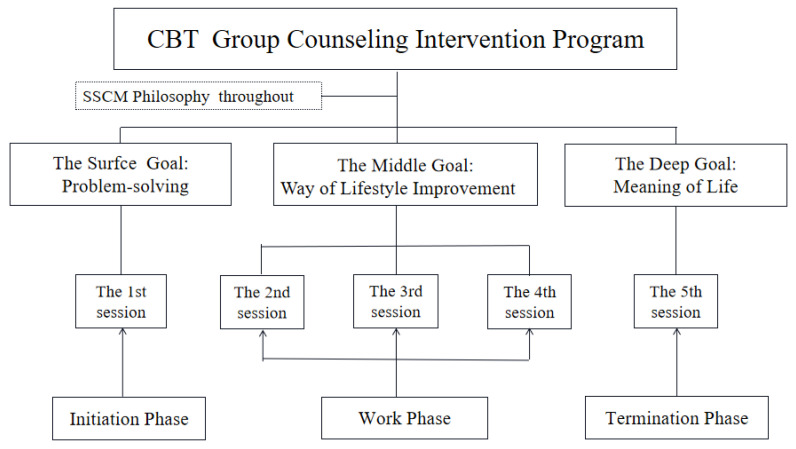
Conceptual diagram of the intervention program.

In summary, the overall design of this intervention program was a group counseling intervention program based on CBT theory, and the SSCM philosophy was consistent throughout. In addition, the specific activities were an integrated intervention approach, with various psychotherapy techniques applied. A conceptual diagram of the intervention program is presented in Figure 2.

#### 2.3.3. Intervention Program Goals

The overall goal of the group counseling was to promote college students’ personal growth, break away from their IA, and better adapt to college life. The expected effect was that in the intervention group, college students would be able to find their superior resources, figure out how to cope with stress, improve their problem-solving skills and lifestyles, and find meaning in life. The frequency and duration of the intervention program was 90 min per session for a total of 5 sessions (7.5 h), delivered centrally over 1 weekend day.

The first session was titled “E-Net Sharing”, and was designed to allow group members to get to know each other, sign group agreements, and share their histories, experiences, resources, and impressions about internet use.

The second session was “Know Yourself”, and the goals were to learn the characteristics of one’s stress disposition and self-care behaviors, recognize one’s predominant resources, and increase one’s sense of self-esteem.

The third session was “Problem Solving”, and the goals were to inspire the wisdom of group members to seek out each other’s superior resources and confront practical concerns.

The fourth session was “Meaning of Life”, and the goals were to get the group members to think deeply about the meaning and values of life through sharing with each other and to increase their sense of meaning.

The fifth session was “True Confessions”, and the goals were to share their insights with each other and process their feelings of separation among group members.

The composition of the intervention program and the general content of the main activities are presented in Table 1.

### 2.4. Measurements

#### 2.4.1. Internet Addiction Tendency

IA tendencies were measured by Average Daily Internet Use Time (1 item) and Young’s Internet Addiction Test (YIAT) [30], which includes 20 items that are rated on a 5-point Likert scale. The total score ranges from 20 to 100, with high scores indicating high levels of IA. The cut-off value of 50 was used to identify IA in the present study [11].

#### 2.4.2. Psychological State Indicators 

Psychological state indicators were measured by the scales as follows.

The K6 [31] is a 6-item short-form screening scale designed to measure psychological distress. This study uses the Chinese version of the K6, which has demonstrated acceptable reliability and validity [32]. Participants were requested to rate how often they felt the following six feelings over the past month: “nervous”, “hopeless”, “restless or fidgety”, “so depressed that nothing can cheer you up”, “everything was an effort”, and “worthless”. Each feeling was rated on a 5-point Likert scale. The total score ranges from 0 to 24, with high scores indicating a high degree of psychological stress. 

Sense of Coherence (SOC) is a global orientation that expresses the extent to which one has pervasive, enduring, and dynamic feelings of confidence, and it is considered part of personal resilience [33]. The participants’ SOC levels were measured using SOC-13 [34], which is the revised version of the 13-item Orientation of Life Questionnaire [33] for the Chinese. SOC-13 has three dimensions (i.e., comprehensibility, manageability, and meaningfulness). Each item was assessed on a 7-point Likert scale. The total score ranges from 13 to 91, with high scores indicating high levels of SOC. 

The Multidimensional Scale of Perceived Social Support (MSPSS) [35] is a self-administered measure of social support. It includes 12 items rated on a 7-point Likert scale measuring three sources of support, namely, Family, Friends, and Significant Other. The total score ranges from 12 to 84, with high scores indicating high levels of perceived social support. 

The General Procrastination Scale (GPS) [36] is a 20-item measure designed to measure procrastination traits across different situations. This study used the Chinese version translated and revised by Chu et al. (2010) [37]. Each item was assessed on a 5-point Likert scale. The total score ranges from 20 to 100, with high scores indicating high levels of procrastination.

#### 2.4.3. Internet Addiction Improvement Motivation 

IA Improvement Motivation was measured by the Internet Addiction Improvement Motivation Scale (IAIMS) [38], which was developed based on a standardized smoking cessation motivation scale to measure motivation to improve IA behaviors. It consists of 10 questions that reflect the characteristics of thoughts and behaviors observed in the first three stages of the five-stage change model: Pre-contemplation (i.e., I do not want to reduce my internet use.), Contemplation (i.e., I have many resources to succeed in reducing my internet use.), and Preparation (i.e., I want to receive professional help to reduce my internet use.). Each item was assessed on a 6-point scale. The total score of each sub-scale was calculated, with high scores in the Contemplation and Preparation indicating high motivation to receive treatment for IA.

#### 2.4.4. Subjective Evaluation of Self-status

Stress (1 item), which measured the degree of stress currently felt by the individual. A higher score from 0 to 10 indicated a higher degree of stress currently being felt. 

Life Satisfaction (1 item), which measured the degree of life satisfaction currently felt by the individual. A higher score from 0 to 10 indicated greater current life satisfaction.

### 2.5. Statistical Analysis

At baseline, we performed independent samples *t*-test between the two groups. To test the intervention effects, we applied analysis of covariance (ANCOVA) with the baseline scores of both groups as input covariates. First, we tested whether the assumptions of the ANCOVA were met for the scores of each item. If the interaction *p* > 0.05 for group x prior scores, ANCOVA was applied, and if *p* < 0.05, ANCOVA was considered unsuitable. To examine the follow-up effects of the intervention program, we conducted one-factor analysis of variance with repeated measures at three time points (pre-intervention, post-intervention, and one month later, respectively) for both groups and performed Bonferroni multiple comparisons for items for which the main effects were significant. The significance level was set at *p* < 0.05, with 0.05 < *p*< 0.10 being considered a significant trend and used as a reference to determine the overall trend. Analysis was performed using SPSS Ver 27.0.

## 3. Results

### 3.1. Participants

The demographic characteristics of both groups are shown in Table 2. In addition, the mean age of the two groups was compared using a *t*-test, and the gender and grade headcount percentages were compared using the χ^2^ test. No significant differences were found. Therefore, random assignment confirmed that participants in both groups were homogeneous in their characteristics at baseline.

### 3.2. Baseline Characteristics

Table 3 shows the mean values of the effectiveness indicators measured at baseline. Significant differences were found between the intervention and control groups at baseline in “Average Daily Internet Use Time” and “Contemplation” of IAIMS (*p* < 0.05). Other variables did not differ significantly between the two groups.

### 3.3. Intervention Effects 

We used analysis of covariance (ANCOVA) to examine changes in scores of the intervention and control groups from pre-intervention to post-intervention. The results showed that the “average daily internet use time” was 5.7 ± 2.2 h at baseline and 5.4 ± 1.3 h afterward for the intervention group, and 7.1 ± 2.1 h at baseline and 7.5 ± 2.9 h afterward for the control group. The interaction *p* = 0.015 (*p* < 0.05) for group x hours of baseline “average daily internet use time” was not suitable for ANCOVA. From paired-sample *t*-tests, no significant differences were found between baseline and posterior scores for both groups (*p* > 0.05). 

Mean YIAT scores for the intervention group were 59.7 ± 8.5 at baseline and 52.3 ± 8.2 at post, while the control group scores were 59.9 ± 6.1 at baseline and 58.8 ± 7.0 at post. Post scores for the intervention group were significantly lower than for the control group (*p* < 0.01, η^2^ = 0.16). 

The intervention group had baseline scores of 56.3 ± 9.6 and post scores of 59.8 ± 9.5 on the SOC-13, while the control group had baseline scores of 54.0 ± 10.1 and post scores of 53.4 ± 9.5. The intervention group’s post scores were significantly higher than the control group (*p* < 0.05, η^2^ = 0.07). 

Post scores for the intervention group showed a significant decreasing trend over the control group on GPS (56.1 ± 9.3 vs. 60.2 ± 11.8, *p* = 0.073, η^2^ = 0.02). 

For the “Preparation” of IAIMS’ scores, the post score for the intervention group was significantly higher than the control group (15.8 ± 2.7 vs.12.9 ± 3.8, *p* < 0.05, η^2^ = 0.08). 

For the “Stress” score, the intervention group’s post score was significantly lower than the control group (6.6 ± 1.7 vs. 7.3 ± 1.3, *p* < 0.05, η^2^ = 0.08). 

No other significant differences were found between the two groups in the K6, MSPSS, Pre-contemplation and Contemplation of IAIMS, and Life Satisfaction (Table 3).

### 3.4. Sustained Effects 

We performed one-factor analysis of variance with repeated measures and performed Bonferroni multiple comparisons for items for which the main effect was significant to examine the sustained effects of the intervention program.

Significant differences in main effects were found in the intervention group for items such as YIAT, MSPSS, and Preparation of IAIMS. For YIAT scores, the main effect of the intervention program was significant at F (2,40) = 7.76, *p* < 0.01. Multiple comparisons showed that scores were significantly lower at the 5% level than before the intervention, both after the intervention and one month later. 

For MSPSS scores, the main effect of the intervention program was significant at F (2,40) = 4.87, *p* < 0.05. Multiple comparisons showed that scores were significantly higher after the intervention than before the intervention at the 5% level. In addition, for the 3 sub-scales of MSPSS, the results showed significant differences in the main effects in the “Friends” (F (2,40) = 3.86, *p* < 0.05) and the “Significant Others” (F (2,40) = 6.68, *p* < 0.01) perception of support. The multiple comparisons showed that it was significantly higher at the 5% level after intervention than before intervention for the “Friends”, and it was significantly higher at the 10% level after the intervention than before the intervention, and significantly higher at the 5% level one month later for the “Significant Others”. 

For the Preparation of IAIMS, the main effect of the intervention program was significant at F (2,40) = 4.26, *p* < 0.05. The multiple comparisons showed that it was significantly higher after the intervention than before the intervention at the 5% level. 

No significant differences were found among the three time points for the other scales based on repeated measurements (Table 4).

In the control group, there were no significant differences among the three time points in repeated measures for any of the scales (Table 5).

## 4. Discussion

Through Randomized Controlled Trial, this study examined the effects of the short-term intensive-type CBT intervention program for Chinese college students with IA. The results suggested that the intervention program may reduce Chinese college students’ IA symptoms and improve their SOC, and have the potential to decrease their tendency to procrastinate. In addition, sustained effects were shown to have the potential to improve students’ tendency toward IA and the perception of social support from significant others. The main results were discussed as follows.

### 4.1. Internet Addiction Tendency

Previous studies on group counseling intervention with CBT [17,22,23] reported a reduction in IA symptoms in the intervention group after receiving the intervention program, and similar results were obtained in the present study. YIAT—the main outcome of this study—was used to measure the three-factor model classified as (a) Withdrawal and Social problems, (b) Time management and Performance, and (c) Reality substitute [39]. “Withdrawal” refers to the difficulties and bad mood one experiences when restricted from using the internet. “Social problems” refer to the use of the internet for social comfort and social interaction as a substitute for real-life social activities. “Time management” refers to compulsive internet use and an inability to control the amount of time one spends online. “Performance” is defined as neglecting one’s studies or work due to a lack of self-control. “Reality substitution” refers to viewing the internet as another reality and using it to avoid real-life problems [39]. Based on the above, we believe that a reduction in IA symptoms means a reduction in the symptoms exhibited by these factors.

Before participating in this study, many participants had a vague sense of possible problems, such as excessive smartphone use, but did not have a proper awareness of IA and its impact on their physical and mental health. In the training course, the participants learned the correct knowledge about IA and became aware of their own IA problems. The main activity of the first session was titled “He/She is in my mind”. The participants directly role-played the smartphones, computers, or internet services that they were most immersed in their daily lives, allowing them to objectively observe their own IA behaviors from the perspective of the object of their addiction and inspiring them to think critically about their relationship with IA. In the second session, through learning self-care behaviors and discussion among group members, the participants developed their own list of effective health behaviors, such as writing, speaking, dancing, singing, drawing, exercising, and making videos, which could replace their IA behaviors. We think it worked directly on the “Reality substitution” factor of IA. The main activity of the third session, called “ Exchange of characters”, helped the participants find solutions to their current issues and improve their self-esteem through role-playing and brainstorming. We think it might be helpful in improving the “Withdrawal and Social problems” factor of IA. The theme of the fourth session was “The Meaning of Life”, which aimed at getting the participants to think about their meaning of life. Since it also included the concept of “Valuing time”, we thought it could be effective for the “Time management and Performance” factor of IA. The main activity of the fifth session was “Looking forward to the Future”, which prompted the participants to own sense of direction and goals for the future. Based on the above, we believe that this intervention program was effective in reducing IA symptoms among Chinese college students. 

### 4.2. Psychological State Indicators 

The intervention improved the participants’ SOC, especially their meaningfulness. Based on the feedback, we think that the main effect came from the fourth session—“The Meaning of Life”. The philosophy of SSCM, which runs throughout the whole intervention program, also had a positive effect. Through the intervention, participants felt respected and understood, which motivated them to believe in their own strengths and innate resources, and inspired them to be willing to embrace a “meaningful” life.

The reduction of participants’ procrastination was attributed to the third session—“Problem Solving”. In this session, the participants selected the “tendency to procrastinate” as a common problem they wanted to solve. Then the group leader used brainstorming to stimulate the potential of group members to find a solution together. We believe this was much more effective than delivering the answer directly.

In terms of sustained effects, the intervention program had a significant effect on improving the participants’ perceptions of social support from “Significant Others”. Meta-analysis of group counseling intervention studies of Chinese college students’ IA showed that the intervention had effects on improving students’ communication skills and interpersonal relationships [17,19], and similar results were observed in the present study. Participants in the intervention group realized that they were not isolated, and each group member had similar problems, which could help them rapidly increase their sense of identity and belonging and present their deeper problems in a safe atmosphere. The support of group members allowed the participants to feel great interpersonal warmth. Furthermore, the mutual imitation and guardianship not only enable their personal growth, but the friendships formed through the activities also have an impact on their real lives. We think this increased their perceived social support from significant others.

### 4.3. Items with No Significant Improvement Effect

The present intervention program did not result in a significant decrease in “Average Daily Internet Use Time” of college students. The internet is an important tool for modern education and entertainment; it is an integral part of college students’ daily life. A previous study suggested that internet use and online gaming had many beneficial effects on individuals [40]. King and Delfabbro (2016) emphasized that even in the case of gaming addiction, “online gaming time” was not equivalent to the dose of substance abuse and could not adequately represent the reality of an individual’s usage [41]. Fhkps and Leung (2013) suggested that complete abstinence was not a viable solution for any intervention [42]. In fact, some participants reported that their internet use time actually increased after the intervention because they needed to use the internet to study English and gather information for writing papers. Therefore, when evaluating the effectiveness of an intervention program, it was important to discern what use should be restricted, rather than only the hours spent online. 

The results of the present study showed that the K6 scale, which measured the psychological distress of college students, had no significant improvement after the intervention and one month later, which was different from the results of previous studies [17,23]. We think the main reason might be related to the timing of the intervention. It was implemented in mid-December 2020, when final examinations were approaching. The tension caused by the exams was high for many college students. Furthermore, the one-month follow-up period was around mid-January 2021, exactly when new waves of COVID-19 spread in mainland China; thus, people became more nervous about being infected. College students had to stay at home, which may not only lead to an increase in their internet use but also had a significant impact on psychological distress. Although the intervention program was designed with a stress management component aimed at improving the psychological distress of college students, its ameliorative effect was proven to be insufficient in the specific context of COVID-19. We should consider enhanced intervention programs in future studies.

### 4.4. Limitations and Future Directions

The first limitation of this study is the potential bias in participants’ motivation. Although randomization was implemented in this study to assign subjects to intervention and control groups, neither participants, implementers, nor evaluators were blinded. Furthermore, when recruiting participants, some applied from a “Recruitment Poster”, while others were recommended by their academic tutors. Therefore, sampling bias may exist. The third limitation is the magnitude of power. Although the 28 participants satisfied the requirement to verify the effectiveness of the intervention program in this randomized controlled trial, the actual sample size of 43 participants was still small, making it difficult to confirm significant differences in the effectiveness in clinical practice, which may reduce the power of the study. Therefore, future studies should increase the number of participants and test intervention effects at multiple universities. The final limitation is the timing of implementation. It was a period of heightened tension over the COVID-19 infection in many countries, and Chinese college students were forced to stay at home, causing them to feel stressed. Due to the need to develop a short-term, intensive intervention program that could be implemented over a weekend for the duration of COVID-19, the five sessions were all important, yet the volume was undeniably enormous to implement all at once over one day. Intervention programs conducted for 8 weeks or longer have been shown to be more effective than interventions of shorter duration [43]. In the future, we think it is necessary to consider a program that provides a certain period of time for systematic intervention with the aim of achieving sufficient sustained effects. Finally, limited by the effects of short-term focused interventions, indicators of the effects of personality traits were not addressed in this study, and we will consider this component in future long-term intervention programs.

### 4.5. Contributions and Implications

Despite some limitations, as noted above, there were several contributions that should not be neglected. Firstly, there were innovations and developments based on the conventional CBT intervention programs. Similar to the conventional CBT interventions, this program attempted to make participants aware of their attitudes toward internet use and IA symptoms through specific activities with the aim of eliminating “cognitive distortions”, and thus improving their problem-solving and adaptation skills. The difference was that instead of simply considering IA as a problem, we helped the college students think about their own lives from a broader perspective, which we thought may help improve their SOC while reducing their IA symptoms. Secondly, this study introduced the SSCM philosophy into a group counseling intervention program for the first time. That was, the group leader did not assume the role of an expert but rather believed in the problem-solving abilities of the participants and respected their own resources [28]. By reaching the three goals of SSCM, the results were expected to have a long-term positive impact on their lives. Thirdly, in addition to CBT and group counseling techniques, this intervention program also incorporated various psychotherapy techniques. These psychotherapies had the common therapeutic perspective of positive psychology and focused on positive resources rather than negative issues, which was consistent with the overall philosophy of this intervention program. This allowed the group leaders to focus on stimulating the group’s resources and creativity without criticizing or blaming the participants, and in an atmosphere where participants felt respected and understood, became aware of their own problems and resources, and that being proactive in finding solutions to problems can be useful in maintaining a positive outlook on their future lives.

## 5. Conclusions

We developed a short-term intensive-type CBT intervention program for Chinese college students with IA. The results showed that the intervention program reduced college students’ internet addiction symptoms and procrastination and improved their sense of coherence. The intervention had sustained effects on internet addiction symptoms and perceived social support among Chinese college students.

## Figures and Tables

**Table 1:** Intervention Program Composition.

Phase	Theme	Composition	The General Content of the Main Activities
Initiation phase	The 1st session E-Net Sharing	Icsebreaker (Name yourself) Role play (He/She is in my mind) Metaphorical story (A pine in Huangshan Mountain)	Role play (He/She is in my mind) Participants were asked to choose one object they are addicted to from smartphone, computer, online game, application, etc., name it, and write a monologue about what are his/her expectations, what kind of journey he/she has been on, and what are his/her feelings … Sign his/her name. Then discussion in the group.
Work phase	The 2nd session Know Yourself	Icebreaker (Chicken evolution) Exercise (Stress temperament coaching) Group feedback	Exercise (Stress temperament coaching) The group leader introduced participants to the characteristics of three kinds of stress temperaments and self-care behaviors. Then, participants shared with group members what they had learned about their own temperaments and stress coping strategies for themselves.
	The 3rd session Problem Solving	Icebreaker (Rotten water in your house) Role play (Exchange of characters) Metaphorical story (Inner power)	Role play (Exchange of characters) Participants were asked to fill out a list of roles and pick the roles they would like to play (i.e., child, old person, wise person, artist, dreamer, perfectionist, parent, clown, hero, etc.). Then write down the problems and challenges they were facing, and draw out three common problems among the group members, using brainstorming, or imagining the personality of their chosen roles, to find solutions to these problems one by one.
	The 4th Session Meaning of Life	Icebreaker (A thousand knots in the heart) Film viewing (“Ikiru”) Group sharing (Before I die...) Metaphorical story (The Life Train)	Film viewing (“Ikiru”) By viewing Japanese film director Akira Kurosawa’s masterpiece “Ikiru,” we explored the following themes. (i) Loneliness, (ii) Fear of death, (iii) Antidote to the fear of death, (iv) Bringing meaning to our lives, (v) Why does death promote growth in life?
Termination phase	The 5th Session True Confessions	Icebreaker (your body) Group Activity (True Confessions) Group Activity (Looking forward future) Review (My harvest)	Group Activities The group members wrote down the strengths they noticed about each other and their blessings for each other. They then formed a circle, and each member expressed what they were looking forward to in the new year and how they wanted to change themselves through gestures and slogans. Before the session ended, they reviewed all the intervention sessions and recorded their insights and impressions.

**Table 2:** Demographic characteristics of the sample (*n* = 43).

Variable		Intervention Group (*n* = 21)	Control Group (*n* = 22)	*p*
Age, Year ± SD		19.5 ± 0.8	19.9 ± 0.8	0.184 ^a^
Gender, *n* (%)	Male	7 (33.3)	6 (27.3)	0.665 ^b^
	Female	14 (66.7)	16 (72.7)	
Grade, *n* (%)	2nd year	17 (81.0)	15 (68.2)	0.576 ^b^
	3rd year	3 (14.3)	6 (27.3)	
	4th year	1 (4.8)	1 (4.5)	

^a^. independent samples *t*-test, ^b^. χ^2^ test.

**Table 3:** Results of the evaluation of the effects before and after the intervention.

Measurements	Baseline		*p* ^a^	Post-Intervention		*p* ^b^	Effect Sizes η^2^
	Intervention Group (*n* = 21)	Control Group (*n* = 22)		Intervention Group (*n* = 21)	Control Group (*n* = 22)		
	Mean (SD)	Mean (SD)		Mean (SD)	Mean (SD)		
IA Tendency							
Internet Use Time	5.7 (2.2)	7.1 (2.1)	0.042 *	5.4 (1.3)	7.5 (2.9)	-	
YIAT	59.7 (8.5)	59.9 (6.1)	0.931	52.3 (8.2)	58.8 (7.0)	0.005 **	0.16
Psychological State Indicators							
K6	6.5 (3.3)	6.1 (2.9)	0.649	5.9 (3.2)	6.5 (4.0)	0.389	
SOC	56.3 (9.6)	54.0 (10.1)	0.451	59.8 (9.5)	53.4 (9.5)	0.048 *	0.07
MSPSS	55.3 (13.5)	54.0 (10.1)	0.715	61.0 (12.1)	57.0 (11.6)	0.223	
GPS	58.0 (9.9)	58.9 (11.6)	0.783	56.1 (9.3)	60.2 (11.8)	0.073 †	0.02
IA Improvement Motivation							
Pre-contemplation	9.1 (3.0)	10.5 (4.2)	0.214	9.1 (3.2)	10.1 (3.8)	0.992	
Contemplation	12.3 (2.8)	10.8 (2.0)	0.048 *	13.2 (2.2)	11.6 (3.2)	0.138	
Preparation	13.7 (2.8)	12.0 (3.9)	0.114	15.8 (2.7)	12.9 (3.8)	0.029 *	0.08
Subjective Evaluation							
Stress	6.9 (2.0)	6.7 (1.5)	0.677	6.6 (1.7)	7.3 (1.3)	0.038 *	0.08
Life Satisfaction	6.8 (1.9)	6.7 (1.7)	0.814	7.7 (1.6)	6.9 (1.9)	0.106	

** *p* < 0.01; * *p* < 0.05; † *p* < 0.10, ^a^ *t*-test, ^b^ analysis of covariance (ANCOVA), IA = Internet Addiction, YIAT = Young’s Internet Addiction Test, SOC = Sense of Coherence, MSPSS = Multidimensional Scale of Perceived Social Support, GPS = General Procrastination Scale.

**Table 4:** Results of Follow-up Effectiveness Evaluation of Intervention Group (*n* = 21).

Measurements	Intervention Group (*n* = 21)			F	Main Effect	Multiple Comparisons
	Pre-Intervention (a)	Post-Intervention (b)	One Month Later (c)			
	Mean (SD)	Mean (SD)	Mean (SD)			
IA Tendency						
IUT	5.8 (2.2)	5.4 (1.3)	5.8 (1.7)	0.55	n.s.	
YIAT	59.7 (8.5)	52.3 (8.2)	52.5 (9.1)	7.76	0.001 **	a > b *, a > c *
Psychological State Indicators						
K6	6.5 (3.3)	5.9 (3.2)	6.6 (3.9)	0.55	n.s.	
SOC	56.3 (9.6)	59.8 (9.5)	58.1 (10.4)	1.32	n.s.	
MSPSS	55.3 (13.5)	61.0 (12.1)	59.5 (14.6)	4.87	0.013 *	a < b *
Family	17.8 (5.5)	19.5 (4.5)	18.9 (5.1)	2.35	n.s.	
Friends	20.0 (4.2)	21.2 (3.9)	20.0 (5.3)	3.86	0.029 *	a < b *
Significant Other	17.5 (5.8)	20.2 (4.5)	20.6 (5.3)	6.68	0.003 **	a < b †,a < c *
GPS	58.0 (9.9)	56.1 (9.3)	56.9 (9.1)	0.94	n.s.	
IA Improvement Motivation						
Pre-contemplation	9.1 (3.0)	9.1 (3.2)	9.0 (2.9)	0.03	n.s.	
Contemplation	12.3 (2.8)	13.2 (2.2)	12.5 (2.3)	1.16	n.s.	
Preparation	13.7 (2.8)	15.8 (2.7)	14.5 (3.5)	4.26	0.021 *	a < b *
Subjective Evaluation						
Stress	6.9 (2.0)	6.6 (1.7)	7.3 (1.4)	2.36	n.s.	
Life Satisfaction	6.8 (1.9)	7.7 (1.6)	7.3 (1.9)	3.35	n.s.	

** *p* < 0.01; * *p* < 0.05; † *p* < 0.10, n.s. = non-significant, IA = Internet Addiction, IUT = Internet Use Time, YIAT = Young’s Internet Addiction Test, SOC = Sense of Coherence, MSPSS = Multidimensional Scale of Perceived Social Support, GPS = General Procrastination Scale.

**Table 5:** Results of Follow-up Effectiveness Evaluation of Control Group (*n* = 22).

Measurements	Control Group (*n* = 22)			F	Main Effect
	Pre-Intervention	Post-Intervention	One Month Later		
	Mean (SD)	Mean (SD)	Mean (SD)		
IA Tendency					
IUT	7.1 (2.1)	7.5 (2.9)	7.4 (2.1)	0.30	n.s.
YIAT	59.9 (6.1)	58.8 (7.0)	58.0 (10.1)	0.79	n.s.
Psychological State Indicators					
K6	6.1 (2.9)	6.5 (4.0)	6.6 (3.6)	0.25	n.s.
SOC	54.0 (10.1)	53.4 (9.5)	55.0 (7.6)	0.40	n.s.
MSPSS	54.0 (10.1)	57.0 (11.6)	56.6 (9.6)	2.10	n.s.
GPS	58.9 (11.6)	60.2 (11.8)	59.0 (11.2)	1.05	n.s.
IA Improvement Motivation					
Pre-contemplation	10.5 (4.2)	10.1 (3.8)	10.1 (3.0)	0.33	n.s.
Contemplation	10.8 (2.0)	11.6 (3.2)	11.4 (2.0)	1.14	n.s.
Preparation	12.0 (3.9)	12.9 (3.8)	13.2 (3.6)	1.35	n.s.
Subjective Evaluation					
Stress	6.7 (1.5)	7.3 (1.3)	7.0 (1.7)	1.65	n.s.
Life Satisfaction	6.7 (1.7)	6.9 (1.9)	7.1 (1.8)	0.80	n.s.

n.s. = non-significant, IA = Internet Addiction, IUT = Internet Use Time, YIAT = Young’s Internet Addiction Test, SOC = Sense of Coherence, MSPSS = Multidimensional Scale of Perceived Social Support, GPS = General Procrastination Scale.

## Data Availability

No new data were created or analyzed in this study. Data sharing is not applicable to this article.
